# The Pathogenesis of HCC Driven by NASH and the Preventive and Therapeutic Effects of Natural Products

**DOI:** 10.3389/fphar.2022.944088

**Published:** 2022-07-07

**Authors:** Gaoxuan Shao, Ying Liu, Lu Lu, Guangtao Zhang, Wenjun Zhou, Tao Wu, Lei Wang, Hanchen Xu, Guang Ji

**Affiliations:** ^1^ Institute of Digestive Diseases, Longhua Hospital, Shanghai University of Traditional Chinese Medicine, Shanghai, China; ^2^ Institute of Interdisciplinary Integrative Biomedical Research, Shanghai University of Traditional Chinese Medicine, Shanghai, China; ^3^ Department of Hepatology, Longhua Hospital, Shanghai University of Traditional Chinese Medicine, Shanghai, China

**Keywords:** nonalcoholic steatohepatitis, hepatocellular carcinoma, natural products, pathogenesis, therapeutic strategies

## Abstract

Nonalcoholic steatohepatitis (NASH) is a clinical syndrome with pathological changes that are similar to those of alcoholic hepatitis without a history of excessive alcohol consumption. It is a specific form of nonalcoholic fatty liver disease (NAFLD) that is characterized by hepatocyte inflammation based on hepatocellular steatosis. Further exacerbation of NASH can lead to cirrhosis, which may then progress to hepatocellular carcinoma (HCC). There is a lack of specific and effective treatments for NASH and NASH-driven HCC, and the mechanisms of the progression of NASH to HCC are unclear. Therefore, there is a need to understand the pathogenesis and progression of these diseases to identify new therapeutic approaches. Currently, an increasing number of studies are focusing on the utility of natural products in NASH, which is likely to be a promising prospect for NASH. This paper reviews the possible mechanisms of the pathogenesis and progression of NASH and NASH-derived HCC, as well as the potential therapeutic role of natural products in NASH and NASH-derived HCC.

## Introduction

Nonalcoholic fatty liver disease (NAFLD) is a chronic liver disease. It progresses from fatty infiltration of the liver, known as nonalcoholic fatty liver (NAFL), to a stage of nonalcoholic steatohepatitis (NASH) with inflammatory cell infiltration and varying degrees of fibrosis (Chalasani et al., 2018). NAFLD may eventually progress to hepatocellular carcinoma (HCC) (Vilar-Gomez et al., 2018; Younes and Bugianesi, 2018). In addition to NAFLD, a number of risk factors have been associated with the development of liver cancer, such as, hepatitis B virus (HBV) and hepatitis C virus (HCV) infection, alcohol abuse, obesity, aflatoxin B1, iron accumulation and diabetes (Chang et al., 2013). The metabolism of acetaldehyde by alcohol leads to genetic mutations that regulate the antioxidant pathway leading to the formation of reactive oxygen species and subsequent DNA damage leading to the development of hepatocellular carcinoma. HBV DNA is integrated into host DNA and the HBx viral protein leads to genetic instability and epigenetic modifications leading to genetic mutations. Replication, through various structural and non-structural proteins, HCV infection occurs through the induction of inflammation, reactive oxygen species, angiogenesis and blockage of apoptotic pathways, culminating in the development of hepatocellular carcinoma (Rawat et al., 2018).

In particular, liver-related mortality (e.g., cirrhosis and HCC) is significantly increased in the NASH population (Musso et al., 2011). Furthermore, the incidence of NASH-related hepatocellular carcinoma (NASH-HCC) or NAFLD-related hepatocellular carcinoma (NAFLD-HCC) has increased significantly worldwide and is becoming the most common cause of liver cancer (Degasperi and Colombo, 2016). NASH-HCC has a worse prognosis than other causes of liver cancer (Argyrou et al., 2017). Despite being an important cause of liver cancer, the exact mechanism by which NASH progresses to HCC remains unknown. Currently, there is a lack of effective therapeutic agents for NASH, and there is a lack of tools and indicators to prevent and monitor the progression of NASH to HCC. Therefore, it is of great practical importance to strengthen the research on the pathogenesis of NASH-HCC and find effective drugs to prevent and treat NASH-HCC.

Natural products, especially monomeric compounds isolated from herbal medicines, are active substances with well-defined molecular formulae and spatial structures. A variety of herbal monomers, such as resveratrol (Hosseini et al., 2020), berberine (Zhang et al., 2019) and curcumin (Lee et al., 2019), have been shown to have hepatoprotective, anti-inflammatory and antioxidant effects that can effectively improve NAFLD. Some drugs have been found to be associated with the inhibition of NASH-HCC progression, and thus, these drugs may be effective in treating NASH-driven HCC. This review focuses on the causal factors associated with the development of NASH and the progression of NASH to hepatocellular carcinoma, particularly those related to metabolism, and the mechanisms of action that are involved. We also highlight natural small molecule compounds that have been shown to play a role in NASH-driven HCC and some natural products that may have therapeutic promise for NASH-HCC.

### Pathogenesis of NASH-Driven HCC

#### Insulin Resistance

Steatosis is the accumulation of fat in hepatocytes and occurs when fat input or synthesis exceeds fat output or degradation (Machado and Cortez-Pinto, 2014). The first step in the development of NAFLD/NASH is the accumulation of fat in the liver. This condition that is often associated with features of metabolic syndrome (MetS), such as obesity, type 2 diabetes, dyslipidemia and hypertension (Younossi et al., 2016). There are currently three main mechanisms that are thought to be the sources of excessive lipid accumulation in the liver. These mechanisms are an increase in visceral adipose tissue (AT) lipolysis, the activation of hepatic *de novo* lipogenesis (DNL) and the consumption of a diet with a high calorie and/or fat content (Donnelly et al., 2005). Triglyceride (TG) is the most obvious type of fat in adiposity (Puri et al., 2007). Donnelly et al. demonstrated that approximately 60% of the triglyceride content of the liver comes from free fatty acid (FFA) influx from AT. Additionally, 26% of the triglyceride content of the liver comes from DNL, and 15% comes from the diet (Donnelly et al., 2005).

Insulin resistance (IR) is the key pathogenic event associated with the development of hepatic steatosis. A state of IR makes AT resistant to the antilipolytic effects of insulin. This leads to the breakdown of TG and the eventual formation of FFAs and glycerol (Schweiger et al., 2017). Thus, the lack of AT lipolytic inhibition is associated with a massive release of FFAs. Then, FFAs can be taken up by the liver and accumulate as TG (Samuel and Shulman, 2012). More importantly, increased adipose tissue FFA efflux is a major mechanism of hepatic steatosis (Donnelly et al., 2005). In the presence of IR, higher insulin levels also regulate hepatic lipid metabolism by increasing TG synthesis (Vatner et al., 2015). In the liver, DNL is a key pathway that promotes lipid storage. The enzyme responsible for DNL is upregulated by insulin and glucose through the action of two transcription factors, sterol regulatory element binding protein 1 (SREBP1) and carbohydrate response element binding protein (ChREBP) (Postic and Girard, 2008). Among the insulin receptors, insulin receptor substrate 2 (IRS-2) can act as a regulator of SREBP-1c when activated, affecting DNL (Schreuder et al., 2008). In a state of IR, IRS-2 is downregulated. Therefore, SREBP-1c is overexpressed, and DNL is upregulated (Stefan et al., 2008). Unusually, activation of ChREBP occurs following an increase in glucose concentration, thereby enhancing hepatic glycolysis. Thus, intermediate and final products of glycolysis are able to activate ChREBP, which regulates the expression enzymes involved in lipogenesis, such as acetyl coenzyme A carboxylases (ACCs) and fatty acid (FA) synthases (Beaven et al., 2013). In addition, β-oxidation of FFAs is inhibited in an insulin-resistant state, which further promotes hepatic lipid accumulation (Postic and Girard, 2008).

In addition to increased efflux of FFAs from AT to the liver and increased hepatic adipogenesis in the liver, exogenous lipids are another important source of hepatic triglycerides. Thus, dietary factors are clearly critical to the development of NAFLD. High fat intake (typical of the so-called Western diet) is associated with IR, dyslipidemia and metabolic/cardiovascular diseases (Fan and Cao, 2013). Studies have found that human subjects with diets rich in saturated fat exhibit increased AT triglyceride stores and increased intrahepatic TG levels (Luukkonen et al., 2018). In addition, chronic consumption of diets containing 45%–68% energy from fat has been reported to increase intrahepatic TG in rodents (Wang et al., 2006; Koppe et al., 2009). In addition, increased intake of sugary foods and beverages has been associated with the development of NAFLD. The levels of TGs derived from adipogenesis have been reported to be elevated in subjects consuming a high-carbohydrate diet (Faeh et al., 2005; Luukkonen et al., 2018). Unlike glucose, fructose can regulate hepatic lipid metabolism by directly activating SREBP-1c and ChREBP and by decreasing mitochondrial β-oxidation. Thus, it ultimately promotes the development of steatosis, as demonstrated in dietary studies conducted in rodents fed a high-fructose diet (Kennedy et al., 2007; Caligiuri et al., 2016). By observing the key role of the Western diet in the development of NAFLD, it was shown that lifestyle changes can greatly improve metabolic abnormalities, hepatic steatosis and inflammation (Wong et al., 2013; Rodríguez-Piñeiro and Johansson, 2015; Romero-Gómez et al., 2017).

IR is also associated with the development of HCC, which has been demonstrated in mouse model of choline-deficient l-amino-acid-defined diet (CDAA)-induced NASH (De Minicis et al., 2014). To overcome IR and maintain normal metabolic functions, insulin secretion increases, resulting in compensatory hyperinsulinemia (Bugianesi et al., 2005). Previous studies have described insulin and insulin-like growth factor (IGF)-1 as growth factors leading to the inhibition of cell proliferation and apoptosis (Prisco et al., 1999). It has been elucidated that hyperinsulinemia can promote the synthesis and biological activity of IGF-1 (Le Roith, 1997). The liver is the source of over 80% of circulating IGF-1, and the primary stimulus for IGF-1 synthesis in the liver is provided by growth hormone (GH) signaling. Insulin upregulates human hepatic GH receptors (Leung et al., 2000). As a result, hyperinsulinemia produces and releases large amounts of IGF-1 from the liver and exerts growth factor-like activity on hepatocytes. This stimulates cell proliferation and inhibits apoptosis, which increases the risk of hepatocellular carcinogenesis. Moreover, insulin receptor substrate (IRS) binds to insulin or IGF to activate phosphatidylinositol 3-kinase (P13K)/protein kinase B (Akt) and mitogen-activated protein kinase (MAPK) to promote HCC occurrence (Weng et al., 2010). Not only does high glucose provide a substrate for energy metabolism in tumor cells, but sustained hyperglycemia leads to a glycosylation reaction that generates advanced glycosylation end products (AGEs). AGEs bind to the receptor for advanced glycosylation end products (RAGE), activating nuclear factor kappa-B (NF-κB) and inflammatory signaling cascades and generating ROS to induce HCC development (Jabir et al., 2018). IR may also directly accelerate hepatocarcinogenesis by stimulating hepatic neovascularization (Kaji et al., 2008).

### Lipotoxicity

Lipotoxicity is defined as the dysregulation of the lipid environment and/or intracellular lipid components resulting in the accumulation of harmful lipids, which are associated with organelle dysfunction, cellular damage and death. Although triglycerides are a major component of hepatic lipids in NASH and simple steatosis, they are a safe storage lipid with little lipotoxicity (Yamaguchi et al., 2007; Wouters et al., 2010). Potential lipotoxic molecules include cholesterol (Musso et al., 2013; Hendrikx et al., 2014; Arguello et al., 2015), FFAs and their derivatives (Neuschwander-Tetri, 2010), diacylglycerols (Kumashiro et al., 2011) and ceramides (Chaurasia and Summers, 2018). There are three general mechanisms by which lipotoxicity causes cellular damage. (1) Harmful lipids alter the biology and function of intracellular organelles, such as the endoplasmic reticulum (ER) and mitochondria. (2) Direct modification of intracellular signaling pathways, such as ceramide enrichment, may regulate metabolic and inflammatory pathways (Perry et al., 2014). (3) Interactions between lipids located on the cell surface or in the cytoplasm and cellular kinases indirectly modify signaling leading to inflammation and other biological effects. For example, palmitate (PA) can activate Toll-like receptor 4 (TLR4) and thus promote inflammation (Lee et al., 2001).

Hepatocyte death caused by lipotoxicity correlates with the severity of NAFLD, and lipotoxicity plays a role in the activation of hepatic macrophages by damaged and dead hepatocytes (Malhi et al., 2006; Cazanave and Gores, 2010). Enhanced lipid peroxidation can activate macrophages by producing ligands for scavenger receptors (e.g., ox-LDL) (Terpstra et al., 2000). Stimulation of TLR4 by saturated fatty acids is another mechanism of macrophage activation that may contribute to the hepatic inflammatory response (Shi et al., 2006). Importantly, macrophage-mediated stimulation of surviving hepatocytes via NF-kB and other cell proliferation pathways is a major component of hepatocarcinogenesis (Maeda et al., 2005; Sun and Karin, 2012).

### ER Stress

In eukaryotes, the ER is an intracellular organelle that supports many important cellular processes, including the folding of membranes and secreted proteins, the synthesis of lipids and sterols, and the storage of free calcium (Gething and Sambrook, 1992; Schröder and Kaufman, 2005). Disturbance of any of these processes exerts stress on the ER and interrupts the protein folding process. Normally, unfolded or misfolded proteins remain in the endoplasmic reticulum and are eventually folded or degraded. However, when the ER is stressed, unfolded or misfolded proteins can accumulate in the lumen of the ER, a condition known as ‘endoplasmic reticulum stress'. To restore homeostasis *in vivo*, the ER triggers a complex adaptive response collectively known as the unfolded protein response (UPR) (Ron and Walter, 2007; Hetz, 2012). The UPR is the first process used to re-establish ER homeostasis to recover from or adapt to ER stress. However, when the persistence of ER stress cannot be blocked or restored, prolonged activation of the UPR triggers the apoptotic pathway. This pathway leads to cell death to eliminate the stressed cells (Zhang and Kaufman, 2008b). The UPR is characterized by the activation of three distinct signaling pathways that are mediated by three transmembrane sensors: protein kinase RNA activation (PKR)-like ER kinase (PERK), inositol requiring enzyme (IRE)1 and activating transcription factor (ATF) 6 (Ron and Walter, 2007; Malhi and Kaufman, 2011). In the absence of ER stress, the three transmembrane UPR sensors remain inactive by binding to ER chaperone binding immunoglobulin protein (BiP) (Hotamisligil, 2010). However, under stressful conditions caused by the accumulation of misfolded or unfolded proteins, depletion of ER calcium levels or increased free cholesterol in the ER lumen, BiP segregates to activate the UPR. This leads to the activation of IRE1-, PERK- or ATF6-mediated signaling pathways (Szegezdi et al., 2006).

All three ER stress-sensing pathways (ATF6α, PERK/eukaryotic translation initiation factor 2α (eIF2α), and IRE1α/X box binding protein 1 (XBP1)) can regulate the development of steatosis in the liver (Rutkowski et al., 2008). Notably, both XBP1 and eIF2α have been shown to be involved in basal and/or diet-induced regulation of lipid metabolism (Lee et al., 2008; Oyadomari et al., 2008). Xiao et al. (Xiao et al., 2013) demonstrated that mice lacking activating transcription factor 4 (ATF4), a major ER stress mediator, were protected from high-fructose diet-induced hepatic steatosis. A study in zebrafish showed that ATF6 deficiency prevented steatosis during chronic ER stress but exacerbated steatosis during acute ER stress. These findings suggest a protective and pathological role for ATF6 in fatty liver disease (Cinaroglu et al., 2011). Another mechanism leading to hepatic steatosis is the reduction of VLDL synthesis and secretion. Packaging of TG into VLDL is a complex process that occurs in the ER lumen and requires the synthesis of apolipoproteins such as apoB100. Studies have shown that ER stress reduces apoB100 levels through the degradation of the PERK-ATF4 branch of the UPR and attenuates apoB100 translation (Qiu et al., 2009). Thus, ER stress can exacerbate hepatic steatosis. In addition, ER stress can also induce hepatic steatosis by increasing the expression of VLDL receptors, and mice lacking VLDL receptors have reduced hepatic steatosis when fed a high-fat diet (Jo et al., 2013). Recently, it has been proposed that ER stress in adipose tissue enhances lipolysis. Hepatic ER stress appears to play an important role in regulating the composition and size of lipid droplets and lipid synthesis, including cholesterol metabolism, through sterol regulatory element binding protein (SREBP) (Hotamisligil, 2010; Zámbó et al., 2013; Nakagawa et al., 2014; Zhang et al., 2014). ER stress in ob/ob mice has been shown to promote SREBP-1c activation and therefore contribute to hepatic adipogenesis. However, the overexpression of the ER chaperone BiP inhibits SREBP-1c activation and SREBP-1c target gene expression (Kammoun et al., 2009), thereby reducing hepatic steatosis.

ER stress induces apoptosis in hepatocytes through three pathways: activation of C/EBP homologous protein (CHOP), C-Jun N-terminal kinase (JNK) signaling and altered calcium homeostasis. Both the PERK and ATF6 pathways induce the activation of CHOP, which is a transcription factor with proapoptotic functions. Additionally, CHOP activation promotes cell death and induces tissue damage (Ron and Walter, 2007; Tabas and Ron, 2011). *In vitro* and animal studies have shown that CHOP deficiency is protective against a variety of pharmacological and physiological injuries (Tabas and Ron, 2011). Other branches of the UPR are also closely associated with cell death. Upon phosphorylation, IRE1 binds the bridging protein TNFα receptor-associated factor 2 (TRAF2) and promotes apoptosis through JNK (Urano et al., 2000) or direct activation of pro-apoptotic molecules, such as Bcl-2-like protein 4 and Bcl-2 homologous antagonists/killers (Hetz et al., 2006). Finally, alterations in calcium homeostasis in the lumen of the ER have been shown to drive ER stress and apoptosis (Zhang et al., 2014).

Finally, ER stress is inextricably linked to the development and progression of liver inflammation. Because it is a key mediator of liver inflammation, the UPR promotes the inflammatory response associated with NASH through the activation of two major inflammatory pathways (JNK)/activator protein 1 (AP1) and IkB kinase (IKK)-NF-κB (Hotamisligil, 2006; Zhang et al., 2006; Zhang and Kaufman, 2008a; Hotamisligil, 2010; Malhi and Kaufman, 2011; Morgan and Liu, 2011; Lee et al., 2012; Lee and Ozcan, 2014; Zhang et al., 2014). NF-kB is a transcription factor that is a major regulator of inflammatory activation, and its IKK2 subunit is a major component required for its activation during the acute inflammatory response (Wullaert et al., 2007). Sustained activation of the NF-κB pathway has been shown in animal models of NAFLD (Cai et al., 2005) as well as in NASH patients (Ribeiro et al., 2004). IKK2 overexpression and sustained activation of NF-kB in hepatocytes leads to a chronic inflammatory state and insulin resistance (Cai et al., 2005). The IRE1 pathway can activate JNK and IKK through their kinase structural domains and interaction with TRAF2, leading to the increased expression of proinflammatory mediators, such as TNFα, IL-1β and IL-6 and NF-κB (Urano et al., 2000; Hu et al., 2006; Zhang and Kaufman, 2008a). In turn, TNFα binding to its receptor can lead to IKK and JNK activation (Aguirre et al., 2000; Wullaert et al., 2006; Meshkani and Adeli, 2009). In addition, IL-1β, IL-6 and TNFα induce the activating element of cyclic AMP response element-binding protein H (CREBH). CREBH is a liver-specific transcription factor that is associated with ER stress. Although ER stress-induced CREBH cleavage does not lead to increased transcription of UPR genes, mature CREBH interacts with ATF-6 to synergistically induce the transcription of many acute phase response genes in the liver, such as C-reactive proteins and serum amyloid P components. Thus, it promotes and worsens the NASH-related systemic inflammatory response (Zhang et al., 2006; Zhang and Kaufman, 2008a; Zhang et al., 2014). ER stress also promotes inflammation through oxidative stress, and the mechanisms of this process are discussed later.

ER stress was identified as a mediator of NAFLD-promoted HCC in one study (Nakagawa et al., 2014). More importantly, spontaneous typical steatohepatitis HCC was observed in these mice even in the absence of carcinogen treatment, suggesting that ER stress is sufficient to induce HCC in the NAFLD setting. This is most likely because enhanced ER stress increases TNF production by macrophages. TNF activates the tumor necrosis factor receptor 1 (TNFR1)-IKKβ-NF-kB pathway in HCC progenitor cells (HcPC) and leads to tumor formation (Nakagawa et al., 2014). The tumor-promoting effect of ER stress was also found in melatonin-accelerated DEN-induced HCC in rats (Moreira et al., 2015). These results suggest that ER stress can act as a trigger for malignant transformation during the progression of NAFLD to HCC. Obesity induces AT macrophages to polarize toward a proinflammatory phenotype (Promrat et al., 2010). These macrophages secrete pro-inflammatory cytokines that, together with excess FFAs, travel toward the liver. In the liver, proinflammatory cytokines and FFAs activate the hepatic macrophage pool. These macrophages, in turn, secrete proinflammatory signals, including the chemokine CCL2. These signals attract monocytes (MOs) from the circulation and thereby maintain liver inflammation (Chalasani et al., 2009). Tumor-associated macrophages (TAMs) contribute to tumor development and metastasis. TAMs secrete cytokines and growth factors and induce cancer stem cell-like properties (Angulo et al., 2007; Malik et al., 2009). Macrophage activation is regulated by several proteins, including IRE1α. IRE1α activation in macrophages is associated with the TLR-mediated production of proinflammatory cytokines and may therefore regulate the inflammatory milieu (Bhala et al., 2011; White et al., 2012). More importantly, IRE1α has been shown to be associated with proinflammatory macrophage polarization (Bhala et al., 2011). Thus, given the role of IRE1α in the inflammatory milieu, IRE1α may also be involved in the formation of specific TAMs in inflammation-induced cancer development.

### Oxidative Stress

Oxidative stress refers to various detrimental processes that are due to an imbalance between the excessive formation of prooxidants (ROS and/or reactive nitrogen species (RNS)) and limited antioxidant defenses (Robertson et al., 2001). This imbalance is common in various pathological processes, and in these processes, oxidative damage leads to cell death and tissue damage (Matés et al., 2008). Mitochondria are not only an important source of intracellular ROS production but also a major target of ROS-induced damage. Thus, they are prone to dysfunction (Turrens, 2003). Moreover, decreased mitochondrial function may lead to enhanced ROS production, which further increases mitochondrial dysfunction (Turrens, 2003; Malhotra and Kaufman, 2007; Zhang, 2010). In NAFLD, there are at least three main mechanisms for the production of mitochondrial ROS. The first mechanism involves the reduction of mitochondrial GSH (Marí et al., 2006). The second mechanism involves the impaired mitochondrial respiratory chain (MRC), and the third involves increased mitochondrial cytochrome P450 2E1 (CYP2E1) levels/activity.

GSH depletion during the UPR drives mitochondrial production of ROS, which leads to cell death. Additionally, the accumulation of unfolded proteins in the ER leads to Ca^2+^ leakage into the cytoplasm, which increases the production of ROS in mitochondria (Malhotra and Kaufman, 2007). Both mechanisms ultimately lead to increased production of ROS in mitochondria. This affects the mitochondrial electron transport chain (ETC) and ultimately affects ATP production. Since protein folding is an ATP-dependent process, it leads to protein misfolding, increases ROS production, and perturbs cellular homeostasis. Therefore, there is clear crosstalk between the ER and mitochondria. CYP2E1 is capable of directly producing ROS. Although its relative contribution to oxidative stress has not been conclusively determined, increased CYP2E1 activity has been observed in NASH patients (Chalasani et al., 2003). Additionally, animal studies using a methionine choline deficient (MCD) diet to induce NASH reported increased CYP2E1 activity in a rat model (Weltman et al., 1996). A recent study using CYP2E1 knockout mice found that CYP2E1 is required for NASH development (Abdelmegeed et al., 2012). The authors noted that high-fat-fed CYP2E1 knockout and WT mice had higher levels of TNF-α in the liver compared to chow (low fat) animals; the levels were lower in CYP2E1 knockouts compared to WT mice (Abdelmegeed et al., 2012). In addition, TNF-α (Day, 2002; Haque and Sanyal, 2002) and lipid peroxidation products (Angulo and Lindor, 2001) inhibit the mitochondrial electron transport chain, damage mitochondrial DNA and deplete mitochondrial cytochrome C (Pessayre et al., 2002). Thus, mitochondrial dysfunction and ROS production are exacerbated. Mitochondrial damage leads to a secondary inhibition of lipid β-oxidation, further increasing steatosis (Day, 2002; Haque and Sanyal, 2002) and initiating a vicious cycle.

Kupffer cells and hepatic stellate cells (HSCs) are nonparenchymal cells that account for almost 40% of all hepatocytes, and they play an important role in the progression of chronic liver inflammation and fibrosis (Rolo et al., 2012). Excessive fatty acid accumulation in hepatocytes causes oxidative stress not only in mitochondria but also in peroxisomes or microsomes. These cytotoxic ROS and lipid peroxidation products can diffuse into the extracellular space, affecting Kupffer cells and HSCs. This cellular oxidative stress from hepatocytes and the direct uptake of free fatty acids or free cholesterol in Kupffer cells induce the activation of NF-κB. NF-κB in turn induces the synthesis of TNF-α and several proinflammatory cytokines, such as IL-6 or IL-8 (Hui et al., 2004). Moreover, inflammation and hepatocellular injury drive the activation of regenerative pathways and the proliferation of fibrotic cells, mainly HSCs. The ability of HSCs to remodel the extracellular matrix (ECM) places them in a strategic position for a microenvironment conducive to cell survival and proliferation. In this environment of continuous regeneration and repair, some hepatocytes may evolve adaptive mechanisms of cell survival and proliferation that promote precancerous transformation and/or tumor growth (Stickel and Hellerbrand, 2010).

### Genetic and Epigenetic Factors

A range of single nucleotide polymorphisms (SNPs) are known to be associated with the presence of NAFLD and the risk of disease progression to advanced fibrosis (Anstee and Day, 2015). Of greatest interest are the genes encoding the patatin-like phospholipase domain-containing protein 3 (PNPLA3; rs738409c.444C > Gp. I148M) and transmembrane 6 superfamily member 2 (TM6SF2. rs58542926c.449C > T, p. E167K), which are strongly associated with steatosis, NASH and the severity of fibrosis or cirrhosis (Romeo et al., 2008; Valenti et al., 2010; Liu et al., 2014a; Kozlitina et al., 2014; Dongiovanni et al., 2015). The PNPLA3 gene is a known determinant of liver fat content, and the PNPLA3 rs738409 C > G SNP leads to the replacement of isoleucine next to the catalytic structural domain with methionine, resulting in the PNPLA3 I148M variant (Banini and Sanyal, 2021). Overexpression of the I148M variant in mouse liver promotes triglyceride accumulation, increases fatty acid synthesis, and impairs triglyceride hydrolysis (Smagris et al., 2015). The I148M variant has also been shown to predispose to HCC in the presence of metabolic risk factors such as T2DM and obesity (Valenti et al., 2013; Ueyama et al., 2016). Patients carrying the PNPLA3 polymorphism not only have a higher risk of steatohepatitis and fibrosis, but also have a more than threefold increased risk of HCC (Liu et al., 2014b; Singal et al., 2014).

Another SNP associated with an increased risk of HCC in NAFLD patients is the TM6SF2 gene. TM6SF2 is thought to be a lipid transporter protein that interacts with proteins involved in intestinal absorption (Mahdessian et al., 2014). Knockdown of TM6SF2 has been shown *in vitro* to reduce secretion of triglyceride-rich lipoproteins and Apo-B (Li BT. et al., 2020). Knockdown of TM6SF2 *in vitro* showed a reduction in secretion of triglyceride-rich lipoproteins and Apo-B (Li BT. et al., 2020). TM6SF2 rs58542926 C > T gene variants leading to substitution of glutamate for lysine result in increased steatosis and advanced fibrosis in NASH patients (Liu et al., 2014a; Kozlitina et al., 2014) and are associated with NASH- HCC (Chen et al., 2015), independent of diabetes, obesity or PNPLA3 genotype. Despite conflicting data on its role in HCC progression, the TM6SF2 variant is thought to be associated with liver injury in the pathogenesis of NASH-associated HCC (Chen et al., 2015). Other genetic abnormalities that may contribute to the development of HCC in NAFLD patients include the C282Y and H63D mutations in the human haemostatic iron regulator (HFE) gene (Bugianesi et al., 2002; Bugianesi et al., 2004; Nelson et al., 2007) and the membrane bound O-acyltransferase structural domain 7 (MBOAT7) gene rs641738 C > T variant (Donati et al., 2017). In particular, the H63D mutation has been identified in non-cirrhotic HCC and leads to liver inflammation, fibrosis and carcinogenesis due to increased iron loading in these patients (Ye et al., 2016). More recently, the rs641738 genotype encoding MBOAT7 has been associated with an increased risk of more severe liver injury and fibrosis in patients with NASH; however, these findings require further investigation into HCC progression (Mancina et al., 2016; Thabet et al., 2017).

Epigenetic changes leading to aberrant DNA methylation are thought to be another important mechanism in the progression of NASH (Iacobazzi et al., 2013). It occurs through the enzyme methyltransferase (DNMT) and leads to gene silencing associated with DNA damage and repair, lipid and glucose metabolism, and fibrosis progression (Tryndyak et al., 2011). In humans, DNMT levels have been found to be higher in NASH patients than in NAFL patients (Pirola et al., 2013). However, the only epigenetic change clearly associated with NASH-related HCC is the gene encoding the chromodomain helicase DNA-binding protein 1 (CHD1) (Liu et al., 2015). NAFLD-related HCC is associated with hypermethylation of the glycine N-methyltransferase (GNMT) promoter, resulting in reduced expression of this gene. GNMT inhibits HCC growth through an unknown mechanism and has been noted to be absent in fast-growing HCC and present at low levels in slow-growing HCC (Simile et al., 2018).

In addition, exome sequencing analysis of HCC revealed the highest mutation rates in oncogenes such as CTNNB1, AXIN1 (involved in the β-catenin/WNT signalling pathway), albumin (ALB), TP53 and CDKN2A (Schulze et al., 2015). In addition, a number of microRNAs (miRNAs) appear to play a role in NAFLD-associated HCC, including miR-21, miR-23, miR-29, miR-93, miR-106, miR-155, miR-221, miR-222 and miR-519 (de Conti et al., 2017). Compelling evidence suggests that miRNA expression is dysregulated in many cancers through a variety of mechanisms and that they may function as oncogenes or tumour suppressors under certain conditions (Erstad et al., 2018). Among these, liver-specific miR-122 expression is reduced in NASH patients and therefore negatively regulates hepatic adipogenesis (Cheung et al., 2008). Downregulation of miR-122 has also been demonstrated in a mouse model of NASH-HCC, suggesting a direct role for this miRNA in NASH-associated HCC (Takaki et al., 2014).

### Gut Microbiota

Under physiological conditions, the gut microbiota regulates hepatic lipogenesis, bile acid metabolism, oxidation and the levels of hepatic inflammatory mediators. In turn, the liver regulates the gut microbiota through the secretion of bile (Jiang et al., 2019). The gut microbiota is thought to be a driver of hepatic steatosis and inflammation (Moschen et al., 2013). Dysbiosis of the gut flora is involved in the development of several liver diseases, including simple steatosis, NASH and NAFLD-HCC. Studies have found dysbiosis of the gut microflora in patients with NAFLD and NASH; these patients have a significantly lower abundance of *Clostridium* than healthy individuals (Mouzaki et al., 2013). The proportion of alcohol-producing bacteria in the gut is also increased in patients with NASH, leading to increased blood ethanol concentrations. Ethanol can induce chronic inflammatory damage to hepatocytes. Therefore, it is likely that alcohol-producing bacteria are involved in the pathogenesis of NASH by participating in oxidative stress and liver inflammation through ethanol metabolism (Zhu et al., 2013). The abundance of *Bacteroides* and Ruminococcaceae were increased in the gut of NASH-HCC patients, and the abundance of *Bifidobacterium* was reduced in these patients (Ponziani et al., 2019). Both *Akkermansia* and *Bifidobacterium* play a protective role against liver injury. Thus, their depletion may enhance inflammation in the gut and liver, thereby promoting the process of NASH-HCC (Fang et al., 2017; Wu et al., 2017).

In addition to changes in the gut microbiota itself, changes in intestinal permeability are also important factors in the development of NASH and NASH-HCC. The intestinal barrier is a multilayered defense system against external pathogens. This barrier includes the intestinal epithelium, the mucus layer, and the immune system associated with the mucosa. These structures maintain a delicate symbiotic balance between the gut-dwelling microbiota and the host—primarily by preventing them from coming into direct contact with each other (Peterson and Artis, 2014). Alterations in gut permeability help activate local immune responses that lead to tissue inflammation and excessive scarring. These alterations provide a source of oxidative stress that promotes the progression from steatosis to steatohepatitis and subsequently to cirrhosis or ultimately to HCC (Arab et al., 2018). In a previous clinical study, NASH patients had higher intestinal permeability than simple steatosis patients and healthy individuals (Luther et al., 2015). Using fecal microbiota transplantation (FMT), the study found that transplanting the microbiome of high-fat diet-fed mice into germ-free recipient mice induced gut barrier disruption, suggesting that gut barrier disruption in NASH is caused by abnormal gut microbiota (Mouries et al., 2019). Increased intestinal permeability is caused by decreased expression of zonula occludens-1 (ZO-1), a representative tight junction protein (Miele et al., 2009; Giorgio et al., 2014; Luther et al., 2015). More importantly, the expression of ZO-1 and junctional adhesion molecule A (JAM-A) were reduced in NAFLD patients (Miele et al., 2009; Rahman et al., 2016).

Studies have found that many bacteria in the gut are associated with the expression of ZO-1. For example, *Lactobacillus*, *Bifidobacterium* spp. and *A. muciniphila* induce ZO-1 expression to promote intestinal barrier integrity (Lam et al., 2012; Ling et al., 2016; Wu et al., 2017). However, *Desulfovibrio* spp. produce genotoxic hydrogen sulfide (H2S), which increases intestinal permeability (Lam et al., 2015). Following disruption of the intestinal barrier, bacteria and pathogen-associated molecular patterns (PAMPs), including lipopolysaccharides (LPS), can be transferred to the liver via the leaky gut. Then, the pathogen recognition receptors are activated, and the immune responses promote the progression of NASH and HCC (Dapito et al., 2012; Chopyk and Grakoui, 2020). LPS-induced liver inflammation occurs through activation of TLR4 in a variety of cell types, including Kupffer cells, hepatocytes, HSCs and liver sinusoidal endothelial cells (LSECs). In Kupffer cells, the activation of TLR4 signaling through myeloid differentiation primary response 88 (MyD88) induces TNF-α and ROS, further enhancing hepatic inflammation. LPS-induced TLR4 production in HSCs induces the production of various chemokines and adhesion molecules, which in turn induce Kupffer cell chemotaxis. Moreover, the activation of TLR4 on hepatocytes induces hepcidin production through the MyD88/c-JNK pathway, which is associated with hepatic lipid accumulation (Kolodziejczyk et al., 2019). In addition to LPS, other PAMPs, including peptidoglycan, flagellin, and bacterial RNA and DNA, can enter the liver due to increased intestinal permeability and trigger an inflammatory response. Activation of TLR9 by bacterial DNA further induces IL-1β production by Kupffer cells, which leads to hepatic steatosis, inflammation and fibrosis (Miura et al., 2010). In addition to TLRs, inflammasome proteins activated and assembled by the nucleotide-binding oligomerization domain (NOD)-like receptor (NLR) recognize PAMPs. This leads to IL-1 and IL-18 production and further inflammation (Szabo and Petrasek, 2015). A previous study showed that the NLR family pyrin domain containing 3 (NLRP3) inflammasome components were significantly increased in NASH patients compared to non-NASH/NAFLD patients (Wree et al., 2014). These results suggest an association between liver inflammation and NLRP3 inflammatory vesicles. PAMPs also activate the HSC senescence-associated secretory phenotype (SASP) and induce epiregulin production through TLRs, further promoting the development of fibrosis (Dapito et al., 2012; Schwabe and Greten, 2020).

Finally, in addition to the gut microbes themselves, another interesting link between the microbiome and NAFLD is microbial metabolites (Zhao M. et al., 2020). Several metabolites, including amino acids, SCFAs, and bile acids, in the gut, circulation, and liver tissues have been identified to promote NAFLD and NAFLD-HCC. In particular, bile acids are closely associated with NASH and NASH-HCC. Bile acids are another important metabolite that link the gut microbiota to liver disease. Bile acids can alter their receptors, the farnesoid X receptor (FXR), to modulate the development of NASH (Jiao et al., 2018). FXR activation is known to reduce triglyceride levels and inhibit fatty acid synthesis and uptake in the liver (Zhu et al., 2011). In addition, a role for FXR in reducing inflammation has emerged (Shaik et al., 2015). The FXR activator obeticholic acid is known to significantly improve fibrosis and disease severity in patients with NASH (Younossi et al., 2019). In general, primary bile acids secreted by the liver are not bound to intestinal microorganisms. Thus, these unbound bile acids are reabsorbed to form secondary bile acids and returned to the liver for detoxification (Jia et al., 2018). Dysregulated bile acid-microbiome crosstalk can impair this process, thereby contributing to inflammation and HCC development. Secondary bile acids have also been shown to regulate immune functions and HCC development. For example, deoxycholic acid (DCA) induces NASH-related HCC by activating mTOR (Yamada et al., 2018). Increased DCA in the liver can also trigger the SASP phenotype in HSCs. This, in turn, leads to the production of proinflammatory cytokines and protumor factors in the liver, thereby promoting HCC (Yamada et al., 2018). Antibiotic treatment reduces the production of DCA-producing bacteria, which in turn reduces the development of HCC (Schwabe and Jobin, 2013). This suggests that the DCA-SASP axis in HSCs promotes the development of obesity-associated HCC. Importantly, blocking DCA production or depleting the gut microbiota may reduce the development of HCC (Ray, 2013).

The role of natural products in NASH-driven HCC:

### Curcumin

One of the bioactive molecules in turmeric is curcumin. Curcumin is a diarylheptane compound with significant anticancer effects due to its interaction with various cell signaling proteins, particularly protein kinases, cytokine signaling receptors, adhesion molecules, and transcription and growth factors (Song et al., 2013). It also exerts potent lipid-regulating, anti-inflammatory, antioxidant and anticancer properties, which are important for the prevention of NASH. The preventive effects of curcumin on NASH have been identified in previous studies. In a methionine choline-deficient (MCD)-induced NASH mouse model, curcumin treatment significantly reduced MCD-induced inflammation but did not affect steatosis or hepatic lipid peroxide levels. In addition, curcumin prevented the activation of NF-kB in these NASH mice (Leclercq et al., 2004), thereby alleviating the progression of NASH. Subsequently, a study using a rabbit NASH model induced by a HFD further confirmed the significant effect of curcumin on the treatment of NASH. It was found that curcumin significantly reduced aminotransferase levels, TNF-α protein levels and mitochondrial reactive oxygen species in rabbits with NASH. Thus, it could increase mitochondrial antioxidant levels and improve mitochondrial function. The investigators suggested that curcumin may regulate NASH through mechanisms involving the antioxidant, anti-inflammatory and mitochondrial protective effects (Ramirez-Tortosa et al., 2009).

More importantly, because of the anticancer effects of curcumin, studies have begun to examine its potential therapeutic role in NASH-HCC. A NASH-HCC model was successfully simulated using streptozotocin (STZ) injections and HFD-fed mice. This model showed 100% reproducibility for hepatocellular carcinoma, and this model demonstrated the progression from fatty liver disease to NASH, liver fibrosis and hepatocellular carcinoma in a diabetic background (Fujii et al., 2013). Interestingly, this study found that the protein expression levels of TLR4 and cytoplasmic translocation of HMGB1 were significantly reduced in the livers of mice in the curcumin-treated NASH group compared to the NASH-only group (Afrin et al., 2017). High-mobility group box 1 (HMGB1) is a DNA-binding nuclear protein, a proinflammatory cytokine without precursors, and an important member of the inflammatory cascade response (Sims et al., 2010). Extracellular HMGB1 can bind to different cell surface receptors, including TLR2, TLR4 and the receptor for advanced glycosylation end products (RAGE), to act on target cells and promote inflammation (Karuppagounder et al., 2015). Activation of these receptors leads to the activation of the NF-κB and extracellular signal-regulated kinase (ERK) signaling pathways, which trigger a variety of inflammatory and pro-inflammatory cytokines. These proinflammatory factors further promote the development and progression of HCC, and curcumin reverses the release of these proinflammatory factors. Moreover, the protein expression levels of SREBP-1c and ADRP, which are associated with DNL, were significantly lower in the NASH-curcumin group. However, Nrf2 expression was significantly higher in the liver, suggesting that curcumin may also enhance the antioxidant capacity of the body (Afrin et al., 2017). However, little research has been done on curcumin, especially in the progression of NASH to HCC, so the specific anticancer mechanism it exerts in the liver in the transition from inflammation to cancer is not well understood. Thus, this will be a focus of subsequent studies.

### Silibinin

Silibinin is derived from milk thistle seeds and is involved in targeting proinflammatory markers as well as reducing lipogenesis (Deep and Agarwal, 2010; Lin et al., 2012; Ghasemi et al., 2013). It has a wide range of pharmacological activities, such as hepatoprotective, antioxidant, and antitumour activities. It maintains hepatocyte membrane stability and is reported to be the most potent flavonoid against liver disease (Muthumani and Prabu, 2012; Bosch-Barrera and Menendez, 2015). Silibinin has been reported to improve dyslipidemia and insulin resistance in HFD-fed Sprague–Dawley (SD) rats by modulating the IRS-1/PI3K/Akt pathway (Zhang et al., 2013). Additionally, silymarin-phospholipid complexes prevented mitochondrial dysfunction in MCD diet-fed Wistar rats (Serviddio et al., 2010), and a mixture of silymarin, phosphatidylcholine and vitamin E has been used clinically to treat NASH patients (Loguercio et al., 2012). Caspase 8 and Fas-related proteins with death domain-like apoptosis regulator (CFLAR) is known to reverse the course of NASH (Wang et al., 2017). Recent studies have shown that CFLAR inhibits the phosphorylation of JNK, thereby ameliorating the pathological features of NASH, such as disorders of glucolipid metabolism, oxidative stress, inflammation and liver fibrosis (Wang et al., 2017). Importantly, silymarin activates CFLAR and inhibits the downstream pJNK/JNK expression *in vivo* and *in vitro*. Furthermore, silymarin has an antagonistic effect on the inhibition of the expression of key genes involved in lipid acid β-oxidation, such as Ppar α, Fabp5, Cpt1α and Acox, that is induced by feeding the MCD diet. These findings suggest that silymarin may promote hepatic fatty acid β-oxidation. Finally, silymarin may also improve NASH-related oxidative stress damage by increasing hepatic antioxidant enzyme activity and inhibiting free radical-generating enzyme activity (Liu Y. et al., 2019). The above findings, especially the modulation of CFLAR by silymarin, provide us with further insight into its potential for the treatment of NASH.

Silymarin has been shown to have anti-inflammatory and antitumour properties. While there are no studies that clearly show that silymarin can halt this progression in NASH-driven liver cancer, similar studies may serve as supporting evidence for this. Previous reports have shown that obesity promotes fatty acid accumulation and inflammation in the liver, leading to liver injury (Marengo et al., 2016). Obesity is associated with an increased risk of liver cancer development and progression (Federico et al., 2015). Serum levels of pro-tumor cytokines and adipokines (e.g., leptin, IL-6, VEGF, IL-8, resistin and endolipin) are higher in obese males than in overweight (OW) and normal weight (NW) males (Steppan et al., 2001; Ramirez et al., 2012). The administration of silymarin significantly inhibited the obesity-induced secretion of MMP-9, a marker of an aggressive phenotype. MMP-9 and obesity further promote the invasive ability of hepatocellular carcinoma cells. The administration of silymarin also significantly reduced ROS production in the OB group and restored these levels to levels close to those of the NW group. Silymarin inhibited obesity-induced ROS production and prevented DNA damage (Miethe et al., 2017). Previous studies have shown that obesity induces adipogenesis, which can lead to steatosis and the progression to hepatocellular carcinoma (Donohoe et al., 2017). Similarly, this study found a significant increase in DNL in OB sera compared to OW and NW sera in hepatocellular carcinoma cells. In addition, the addition of silymarin to OB serum had a significant protective effect on DNL. These data suggest that silymarin may prevent the accumulation of fatty acids in the liver, thereby preventing local inflammation and tumor growth (Miethe et al., 2017). While we cannot be sure whether obesity-induced liver cancer is NASH-driven liver cancer, there are many similarities. Therefore, further validation in animal models is needed.

### Berberine

Berberine is an isoquinoline quaternary alkaloid that is derived from Coptis chinensis and has been reported to be an effective drug with anti-inflammatory and antitumour pharmacological effects (Sun et al., 2009). In previous studies, berberine was shown to ameliorate high-fat, high-cholesterol (HFHC) diet-induced NASH in APOE−/− mice (Yang et al., 2017). The latest study found that berberine also had a therapeutic effect in NASH-HCC mice. This study used STZ injections in combination with a HFHC diet to establish a mouse model of NASH-HCC (Fujii et al., 2013). The model was able to reflect the pathological progression from fatty liver, steatohepatitis and fibrosis to HCC. Phellodendron was found to significantly reduce TC, LDL and HDL levels in NASH-HCC mice, suggesting that berberine could lower cholesterol and lipid levels. Moreover, increased expression of adipogenic genes, such as FASN, SCD1 and SREBP-1c, in STZ-HFHC mice was significantly inhibited by berberine, suggesting that berberine may attenuate lipid accumulation (Luo Y. et al., 2019). Previous studies have shown that berberine can inhibit the overexpression of fibrotic genes, such as α-SMA, fibronectin, and collagen I and III (Chitra et al., 2015). In addition, berberine has been shown to improve liver fibrosis by inhibiting hepatic oxidative stress and the fibrotic potential (Domitrović et al., 2013). Berberine was also found to inhibit the activation of blast cells in the liver of NASH-HCC mice, thereby exerting an antitumour effect. Increased expression of COX2 in NASH-HCC mice was inhibited by berberine, possibly because berberine inhibits inflammation and angiogenesis via the p38MAPK/ERK-COX2 pathway (Luo Y. et al., 2019). It is known that abnormally elevated COX2 levels are associated with inflammation, angiogenesis and the promotion of tumor formation (Möbius et al., 2005; Harris, 2007). In another model of NASH-HCC induced by deoxycholic acid, COX2 overexpression was detected and exerted a protumor effect by suppressing antitumor immunity (Loo et al., 2017). However, given the clinical application of berberine, the elevated COX2 levels in NASH-HCC mice may be due to changes in the intestinal flora that are induced by the HFHC diet. Previous studies have mentioned the effect of berberine on NASH via the gut-liver axis (Cao et al., 2016). Berberine may affect the intestinal microbiota and thus have multiple effects in NASH-HCC. Therefore, further research on this topic is needed.

### Green Tea Catechin

The results from a population-based case–control study suggest that long-term green tea consumption is associated with a lower risk of HCC (Li et al., 2011). These benefits may be attributed to epigallocatechin gallate (EGCG), the major catechin in green tea. EGCG selectively induces apoptosis and cell cycle arrest in HCC cell lines but not in noncancerous hepatocytes (Zhang et al., 2015). In addition, catechin-rich green tea extract (GTE) has been shown to protect obese mice from NASH by inhibiting liver inflammation. This finding is consistent with a mechanism involving the gut-liver axis that limits metabolic endotoxemia and hepatic TLR4/NFκB activation (Li et al., 2018). Many *in vitro* studies have demonstrated the chemopreventive and antitumour properties of green tea in hepatocellular carcinoma (Darvesh and Bishayee, 2013). EGCG inhibits the growth and proliferation of hepatocellular carcinoma cells by inducing apoptosis (Kuo and Lin, 2003). EGCG also inhibited the growth of the human hepatoma cell line HepG2 by inhibiting the phosphorylation of insulin-like growth factor-1 receptor (IGF-1R) and reducing the activation of its signaling molecules, such as ERK, STAT-3, Akt and GSK-3β (Shimizu et al., 2008). However, it is not clear whether its reported anti-inflammatory activity also prevents NASH-induced HCC progression. A recent study found that in a NASH-HCC model (Wang et al., 2009) established by the administration of DEN to mice fed a HFD, GTE inhibited the signal transducer (gp130, tumor suppressor M) that promotes STAT3 activation (Stephens and Elks, 2017). It also downregulated STAT3-dependent genes (c-Fos, c-Myc, survivin) that promote tumorigenesis (Dey et al., 2019). DEN effectively induces fibrosis and hepatocyte proliferation in a STAT3-dependent manner (Ding et al., 2017). Therefore, we have reason to believe that GTE prevents NASH-related HCC in a STAT3-dependent manner, but this requires further study.

The role of potential natural products in NASH-driven HCC:

### Regulating Lipid Metabolism

Changes in lipid metabolism affect the progression of many tumor cells by affecting the processes of cell growth, proliferation, differentiation and migration (Santos and Schulze, 2012; Luo X. et al., 2019). Many natural compounds have been reported to regulate lipid metabolism, particularly through the expression of adipogenesis-related genes. Luteolin (1), tomatidine (2), oxymatrine (3) and oleanolic acid (4) have been reported to regulate glucose homeostasis and lipid synthesis by reducing SREBP-1c, ACC and FAS expression (Liu et al., 2011; Gamede et al., 2019; Kusu et al., 2019; Zhang et al., 2020). Betaine (5) significantly improved hepatic steatosis in C57BL/6J mice by activating AMPK and downregulating SREBP-1c (Xu et al., 2018). It also attenuated hepatic triglyceride accumulation by reducing methylation of the PPARα promoter and upregulating PPARα expression (Wang et al., 2013). Nuciferine (6) treatment essentially reversed the HFD-induced increase in hepatic protein levels of SREBP-1c and fatty acid synthase (FASN), suggesting that nuciferine could attenuate the development of HFD-induced hepatic steatosis (Guo et al., 2013). In addition, baicalein (7) partially reduced methionine and choline-deficient diet-induced hepatic lipid accumulation in mice by modulating the expression of SREBP-1 c, FAS, PPARα and CPT1a (Zhang et al., 2018). Puerarin (8) inhibits FAS and activates AMPK expression, thereby preventing SREBP-1c translocation to the nucleus. Thus, it ultimately reduces lipid synthesis and increases fatty acid oxidation (Kang et al., 2015). Emodin (9) can improve hepatic steatosis in rats with liquid fructose-induced NAFLD by inhibiting the adipogenic transcription factor SREBP-1c and its downstream proteins FAS and stearoyl coenzyme A desaturase 1 (SCD1) (Li et al., 2016). In addition to affecting DNL, some natural products are thought to enhance fatty acid oxidation and thus reduce NASH. For example, nordihydroguaiaretic acid (10) or schizandrin A (11) supplements may reduce obesity, in part by increasing fatty acid oxidation (Li et al., 2016; Jeong et al., 2019).

### Regulating Inflammation

Inflammation is a very important manifestation of NASH and is thought to be an important factor in the progression of NASH to HCC. The discovery of the anti-inflammatory effects of natural products is therefore necessary and is likely to be an important tool in the treatment of NASH and NASH-HCC. Resveratrol (12) can reduce the secretion and expression of proinflammatory cytokines (Cheng et al., 2019). Further studies have shown that resveratrol improves hepatic steatosis by activating the AMPKα-SIRT1 pathway to enhance AMPKα phosphorylation and inhibit the NF-κB inflammatory pathway (Tian et al., 2016). Celastrol (13) significantly reduced TG accumulation in steatotic HepG2 cells and downregulated the expression levels of TLR4, MyD88, phosphorylated-NF-κBp65, TNF-α and IL-1β. In addition, the combination of celastrol and TLR4 siRNA further attenuated the expression of inflammatory mediators. This finding suggests that celastrol may exert a protective effect by inhibiting TLR4-mediated inflammatory responses (Han et al., 2018). Nuciferine (6) ameliorates NAFLD by reducing necroinflammation (IL-1β, IL-6 and TNF-α) and reversing serum markers of MS (TC, TG and LDL-c) (Cui et al., 2020). Emodin (9) downregulates serum IL-1 and TNF-α secretion and exerts anti-NAFLD effects by inhibiting the hepatic TLR4 signaling pathway (Tao et al., 2017). Emodin also inhibits proinflammatory cytokine expression in LPS-induced macrophages by suppressing ERK1/2 and p38 signaling. Thus it reduces inflammatory cell infiltration in the liver and impairs liver function (Jia et al., 2014).

Isoorientin (14) (a flavonoid isolated from several edible plants) significantly improved inflammation by enhancing antioxidant enzyme activity and inhibiting the secretion of inflammatory cytokines (TNF-α, IL-1, IL-6) in high fructose-induced obesity in mice. Thus, it suppressed hyperlipidemia and liver injury (Yuan et al., 2016). Geraniol (15), a monoterpene alcohol isolated from rose and lemon essential oils, prevented increases in serum IL-1β and TNF-α levels and hepatic nitric oxide levels and modulated fructose-induced inflammation and oxidative stress (Ibrahim et al., 2015). Astaxanthin (16), a lutein-like carotenoid isolated from *Haemococcus pluvialis*, has been reported to inhibit the phosphorylation of JNK1 and IκB-β, ROS production and the nuclear translocation of NF-κB p65 in the liver. Thus, it can reduce inflammation in mice fed a high-fructose diet (Bhuvaneswari et al., 2014). In the NASH mouse model, treatment with schisandrin B (17) or kukoamine B (18) downregulated TNF-α, IL-1β and IL-6 levels (Leong et al., 2016; Zhao Q. et al., 2020). Geraniol (15) or betaine (5) supplements have shown anti-inflammatory properties by improving mitochondrial dysfunction in rats and mice fed an MCD diet (Chen et al., 2016). Genistein (19) is a phytoestrogen that is mainly found in soy and has antioxidant properties (Chen et al., 2016). Genistein (19) treatment reduced liver inflammation and fibrosis through a decrease in TNF-α and IL-1β levels in mice with MCD diet-induced NASH (Yoo et al., 2015). Similarly, naringenin (20) and scopolamine (21) were found to significantly inhibit liver inflammation in MCD-fed mice (Liu et al., 2020; Wang et al., 2020).

### Regulating Fibrosis

Liver fibrosis is a major determinant of clinical disease progression and outcome in patients with NASH (Angulo et al., 2015). When excessive fibrosis occurs, the liver structure is inevitably damaged, and fibrosis usually indicates a higher likelihood of progression to cirrhosis and other end-stage liver diseases in NASH (Angulo et al., 2015). The fibrotic process in the liver is thought to be regulated primarily by HSCs, a type of hepatic progenitor cell that is in a quiescent state under physiological conditions (Tsuchida and Friedman, 2017). If liver injury is not resolved, the continued supply of inflammatory cytokines and apoptotic cell bodies will perpetuate the fibrotic effects of HSC and promote further tissue remodelling (Bachem et al., 1992). The disease has formally progressed from NASH to cirrhosis when collagen deposits are present in the majority of liver tissue (Kleiner et al., 2005). Furthermore, results from longitudinal studies have shown that patients with NAFLD/NASH who have severe liver fibrosis are at increased risk of HCC and mortality compared to patients with mild liver fibrosis (Ekstedt et al., 2015; Alexander et al., 2019). However, a significant number of patients with NAFLD-associated HCC (more than one third) are reported to have no extensive fibrosis at the time of presentation (Baffy et al., 2012). An interesting conceptual possibility is the malignant transformation of hepatocellular adenomas (HCA) in non-cirrhotic NAFLD patients. A correlation between malignant transformation of HCA and the metabolic syndrome has been described in a series of US patients (Farges et al., 2011). These data suggest that NAFLD, a hepatic manifestation of the metabolic syndrome, may lead to the development of HCC in the absence of cirrhotic HCA. In addition, inflammatory HCA, a subtype associated with an increased risk of malignant transformation, is associated with obesity, further supporting this association (Chang et al., 2013). However additional epidemiological and pathophysiological data are needed to demonstrate this correlation.

As described earlier, although liver fibrosis is not necessarily a necessary process for the progression of NASH to HCC, once excessive fibrosis has occurred, the liver structure will inevitably be damaged. Therefore, it is particularly important to prevent and control the development of liver fibrosis. Isorhamnetin (22) treatment showed a protective effect against fibrosis by reducing the expression of fibrosis markers in mice with steatosis induced by a combination of a HFD and CCl4 (Ganbold et al., 2019). Astragaloside (23) is the main active component of Radix Astragali that significantly ameliorated liver fibrosis by inhibiting the TGFβ1/Smad pathway in a CCl4-induced mouse model of fibrosis (Yuan et al., 2018). Astragaloside has been shown to have a variety of pharmacological activities, including antioxidative stress, anti-inflammatory, anticancer and anti-fibrotic activities (Rajput et al., 2011; Gui et al., 2013). Salvianolic acid B (SalB) is a polyphenolic antioxidant component extracted from Salvia miltiorrhiza (Zhai et al., 2019). SalB (24) can inhibit NAFLD/NASH by regulating cellular oxidative stress and lipid metabolism and exerting anti-inflammatory and anti-fibrosis effects to improve liver fibrosis (Hong et al., 2017). Pycnogenol (PYC) is a group of flavonoids isolated from French maritime pine bark (*Pinus pinaster* Aiton, synonym *Pinus maritima* Mill.) and is a mixture of proanthocyanidins with potent antioxidant and ROS scavenging properties. PYC (25) improved fibrosis and cirrhosis in NASH rats, and these results suggest that PYC delayed the progression of fatty liver to fibrosis (Mei et al., 2012). Calycosin (26) significantly reduced liver fibrosis in NASH mice by activating FXR and inhibiting HSC activation (Duan et al., 2017). In MCD diet-fed mice, glycyrrhetinic acid (27) exerted several hepatoprotective effects, including improving inflammation and regulating lipid metabolism, in addition to limiting pericellular fibrosis and collagen deposition (Wang et al., 2016). Xanthohumol (28) is a promising compound for the treatment of fibrosis in NASH. It induces the apoptosis of activated HSCs *in vitro* in a dose-dependent manner without impairing the viability of human primary hepatocytes at high doses (Dorn et al., 2010). Thymoquinone (29) has the potential to ameliorate hepatic steatosis, inflammation, apoptotic state and fibrosis in rats fed a HFHC diet (Awad et al., 2016)[136]. Furthermore, in a mouse model induced by an MCD diet, isochlorogenic acid B (30) exhibited a comprehensive antifibrotic effect by downregulating genes involved in fibrosis and inhibiting several profibrotic factors (Liu X. et al., 2019).

### Regulating the Gut Microbiota

The key role of the gut microbiota in NASH and NASH-HCC has been discussed previously. Although the exact mechanisms by which the gut microbiota plays a role in both conditions are currently unknown, studies have identified natural products that may inhibit the development of NASH by modulating gut microbiota. Thus, they have the potential to hinder the progression of NASH to HCC. Salidroside (31) reduced hepatic lipid accumulation, the inflammatory response and insulin resistance in NAFLD rats (Li et al., 2017). In an HFD-induced NASH model in mice, salidroside (31) treatment significantly improved HFD-induced intestinal bacterial and bile acid disturbances, thus demonstrating the potential role of salidroside (31) in NASH treatment via the intestinal microbial-bile acid-FXR axis (Li H. et al., 2020). In addition, resveratrol (12) has been reported to maintain intestinal barrier integrity and limit intestinal inflammation. Treatment with resveratrol (12) resulted in reduced metabolic endotoxemia and altered the intestinal microbial distribution in SD rats fed a HFD (Chen et al., 2020). In a mouse model of HFD-induced NAFLD, quercetin (32) was shown to reverse intestinal flora imbalance and inhibit endotoxemia-mediated TLR4 signaling. Thus, it was able to suppress the inflammatory response and activate reticulo-stress pathways, which ultimately prevented the dysregulation of lipid metabolism-related gene expression (Porras et al., 2017). A subsequent study reaffirmed that the regulation of the gut microbiota and transplantation of gut microbiota by quercetin (32) can influence the development of NAFLD (Porras et al., 2019). This suggests that the prebiotic capacity of quercetin and the gut microbiota transplantation could be used as a protective strategy against obesity-associated NAFLD.

## Conclusion and Perspectives

**FIGURE 1:**
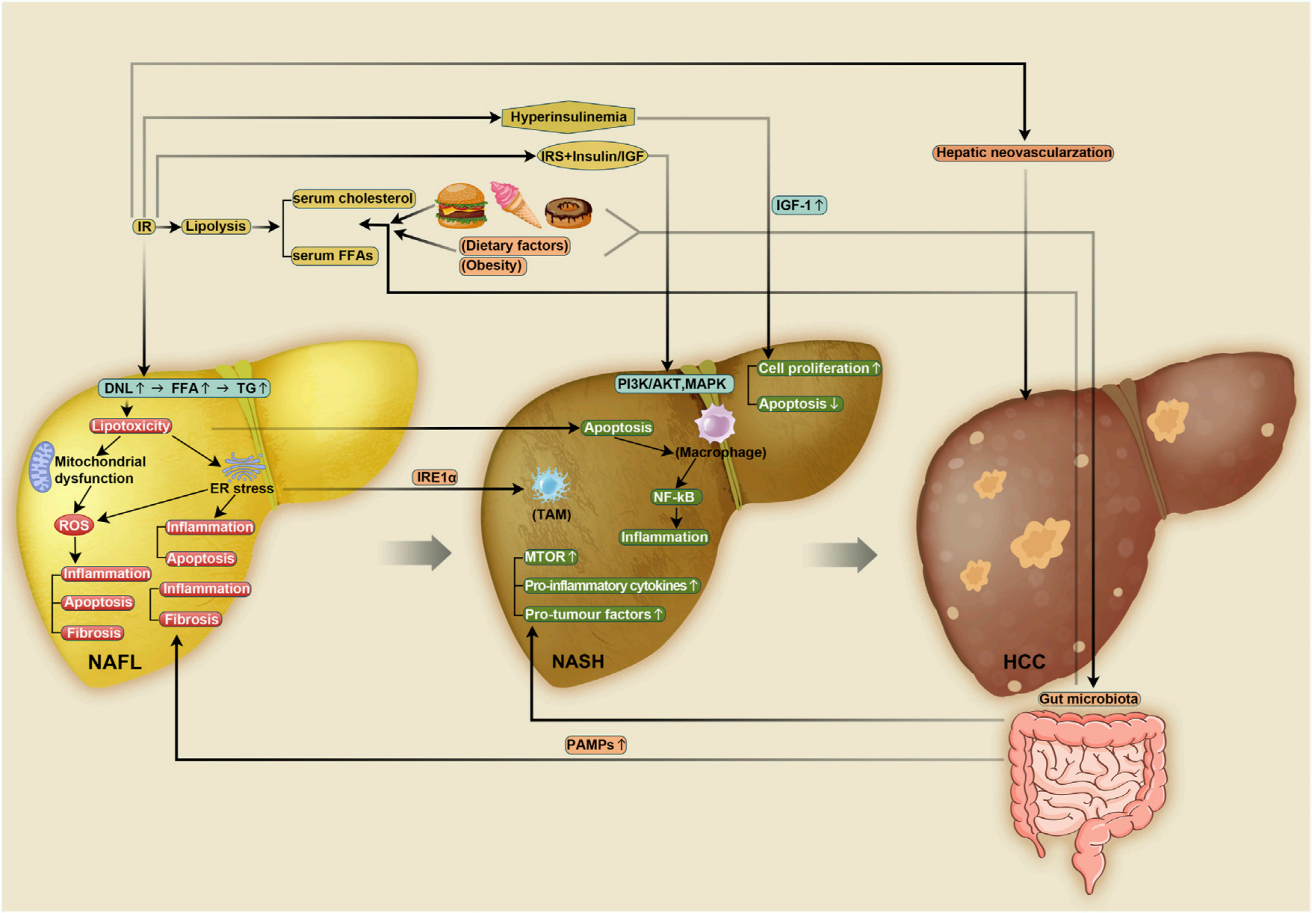
Proposed mechanism of NASH and NASH-driven HCC. Insulin resistance causes a compensatory increase in insulin in the body, leading to hyperinsulinemia and a marked increase in IGF-1 levels. These conditions can promote hepatocyte proliferation and inhibit apoptosis. Therefore, they can exacerbate the development of HCC. Insulin resistance also directly triggers HCC by promoting hepatic neovascularization. When insulin resistance occurs, IRS binds to insulin or IGF and induces HCC through the PI3K/Akt and MAPK pathways. Insulin resistance promotes lipolysis in adipose tissue, which leads to increased serum cholesterol and FFA levels. High levels of FFA in the serum travel to the liver, causing a significant increase in FFA in the liver. Insulin resistance can also directly promote DNL, which leads to an increase in hepatic FFA levels. Increased FFA levels in the liver promotes the synthesis of TG and triggers lipotoxicity. Lipotoxicity induces apoptosis, which activates liver macrophages and triggers inflammation through the NF-kB pathway, further inducing the development of HCC. Lipotoxicity also leads to mitochondrial dysfunction and ER stress, both of which further induce ROS production. This further promotes inflammation, apoptosis and fibrosis, thereby contributing to the development of NASH and NASH-HCC. ER stress can also directly promote inflammation and apoptosis. Dietary factors, obesity and the gut microbiota also contribute to elevated serum cholesterol and FFA levels, and diet and obesity alter the gut microbiota. The gut microbiota promotes inflammation and fibrosis through PAMPs, which further induce NASH and NASH-HCC. Changes in the gut microbiota can also directly trigger HCC through elevated levels of MTOR, proinflammatory factors and protumor factors. IGF: insulin-like growth factor, HCC: hepatocellular carcinoma, PI3K: phosphatidylinositol 3-kinase, Akt: protein kinase B, MAPK: mitogen-activated protein kinase, FFA: free fatty acid, DNL: *de novo* lipogenesis, TG: triglyceride, NF-kB: nuclear factor-jB kinase-b, ER: endoplasmic reticulum, ROS: reactive oxygen species, NASH: nonalcoholic steatohepatitis, PAMPs: pathogen-associated molecular patterns, MTOR: mammalian target of rapamycin.

Based on our account above, it is clear that the current research is primarily focused on NASH. However, there is little research on the progression of NASH to HCC, in terms of both the specific mechanisms involved in the transformation process and its prevention or treatment. The development of NAFLD is clearly associated with metabolic dysregulation as a result of excess carbohydrates and fat leading to insulin resistance, obesity and fatty acid accumulation. These processes upregulate various pathways, including lipotoxicity, oxidative stress, ER stress and innate immune responses, among others. More importantly, a growing number of studies have found that changes in the gut microbiota also play a large role in the development and progression of NASH. The chronic inflammation, regeneration and fibrotic remodeling that follows this predisposes hepatocytes to cellular transformation and chromosomal aberrations, leading to hepatocarcinogenesis (Figure 1). We focused on the process of NASH-driven HCC because the transition from inflammation to cancer is common and can be actively intervened upon to delay or even hinder the development of cancer. Gastritis and colorectal inflammation, if left unchecked, can progress to gastric and colorectal cancer, as it does in the liver. The importance of liver inflammation in the development and progression of cancer has been well documented.

**FIGURE 2:**
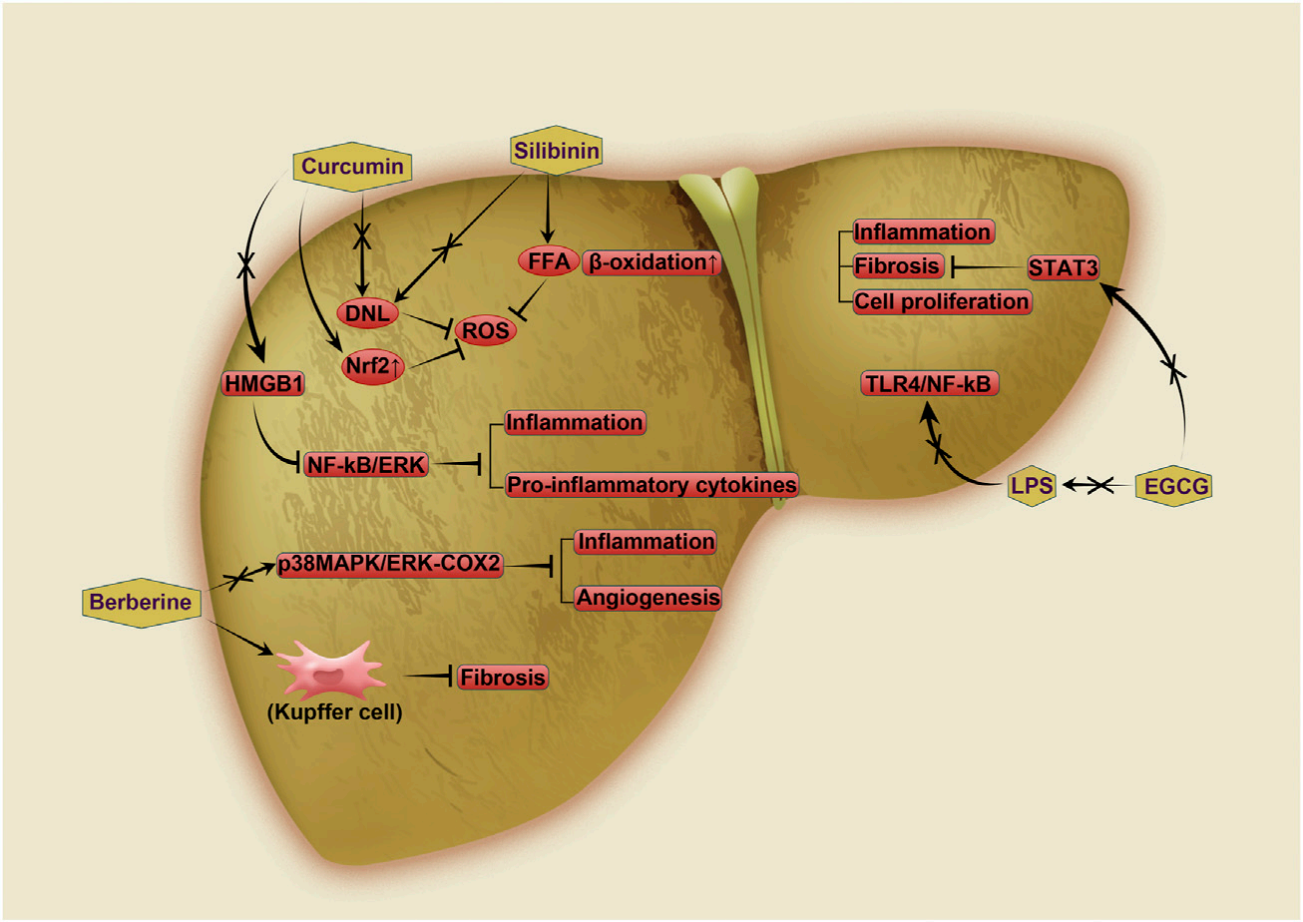
Mechanisms of inhibition of NASH-driven HCC by four natural products. Curcumin can reduce the expression of HMGB1. This can in turn reduce inflammation and the production of proinflammatory factors by inhibiting both the NF-kB and ERK pathways, thereby inhibiting the development of HCC. Curcumin also inhibits NASH and NASH-HCC by downregulating DNL and upregulating Nrf2 expression, respectively, thereby suppressing ROS production. Silibinin also inhibits DNL and, importantly, enhances β-oxidation of FFAs, thereby reducing ROS production. Berberine directly attenuates ROS production and hinders the development of HCC by upregulating p38MAPK/ERK-COX2. Thus, it reduces inflammation and inhibits hepatocyte apoptosis. EGCG regulates the development of NASH-HCC in two ways. First, it inhibits the progression of NASH to HCC by reducing the level of LPS in the blood, thereby inhibiting the TLR4/NF-kB pathway. Additionally, it inhibits HCC development by reducing STAT3 expression and suppressing inflammation, fibrosis and cell proliferation. HMGB1: high-mobility group box 1, Nf-kB: nuclear factor-jB kinase-b, ERK: extracellular signal-regulated kinase, HCC: hepatocellular carcinoma, NASH: nonalcoholic steatohepatitis, DNL: *de novo* lipogenesis, Nrf2: nuclear factor erythroid 2-related 2, ROS: reactive oxygen species, MAPK: mitogen-activated protein kinase, COX2: cyclooxygenase 2, EGCG: epigallocatechin gallate, TLR4: toll-like receptor 4, STAT3: signal transducer and activator of transcription 3.

This review highlights several natural products that have been shown to inhibit the conversion of NASH to HCC, as well as a wide range of natural compounds with great potential to play a role in this process (Table 1). Natural products are derived from components or metabolites in plants and animals and play an important role in the exploration of new structural compounds and drug discovery. The use of plant products for the treatment of cancer has a long history; approximately 60% of the anticancer agents currently in use are of natural origin. Herbs and their derived plant compounds are useful in cancer treatment. Due to their low toxicity, easy availability and low cost, natural compounds are a possible therapeutic agent for the field of anticancer research. An increasing number of natural products have been found to act on NASH. More importantly, research is beginning to focus on the role of natural products in inhibiting the conversion of NASH to HCC. Silymarin, berberine, curcumin and green tea catechin are four natural small molecule compounds that have been shown to impede the conversion of NASH to HCC (Figure 2), although the exact mechanisms of their roles in this process requires further investigation. An increasing number of studies have focused on the effects of natural products on the development of NASH. These effects may act mainly in the following ways: modulating lipid metabolism, anti-inflammatory activities, anti-fibrotic activities, and modulating the gut microbiota. These processes can combat NASH and impede its subsequent progression. Disturbances in lipid metabolism, inflammation, fibrosis and changes in the gut microbiota are all potential factors contributing to the progression of NASH to HCC. It is therefore reasonable to assume that these natural small molecule compounds are likely to be effective drugs for the treatment of NASH-HCC in the future. However, the hepatotoxicity of Chinese herbal medicines has always been a concern. Therefore, while studying the inhibition of HCC by natural products derived from Chinese herbal medicines, we also need to pay attention to the toxicity of HCC. In addition to obtaining evidence of efficacy, comprehensive studies on the genotoxicity, carcinogenicity, and systemic and reproductive toxicity of candidate anti-HCC drugs are urgently needed.

**TABLE 1:** Potential natural compounds with anti-NASH-driven HCC effects.

Biological function	Number	Compound	Model	Ref
Regulating lipid metabolism	(1)	Luteolin	palmitate-stimulated HepG2 cells	Liu et al. (2011)
	(2)	Tomatidine	High-fat and high-fructose diet (HFDHFr)-fed rats	Zhang et al. (2020)
	(3)	Oxymatrine	palmitate-stimulated HepG2 cells	Kusu et al. (2019)
	(4)	Oleanolic acid	High-fat and high-cholesterol (HFHC) diet-fed rats	Gamede et al. (2019)
	(5)	Betaine	AIN-93 G diet-fed ApoE−/− mice	(Wang et al., 2013)
	(6)	Nuciferine	HFD-fed mice	(Guo et al., 2013; Cui et al., 2020)
	(7)	Baicalein	Methionine and choline-deficient (MCD) diet-fed mice	Zhang et al. (2018)
	(8)	Puerarin	Oleic acid (OA)-treated HepG2 cells	Kang et al. (2015)
	(9)	Emodin	fructose-fed rats; LPS-induced acute liver injury (ALI) Balb/c mice; western-type diet-fed LDLR^−/−^ mice	(Jia et al., 2014; Li et al., 2016; Ding et al., 2018)
	(10)	Nordihydroguaiaretic acid	American Lifestyle-Induced Obesity Syndrome (ALIOS) diet-induced mice	Chan et al. (2018)
	(11)	Schizandrin A	High-fat and high-cholesterol (HFHC) diet-fed mice	Jeong et al. (2019)
Regulating inflammation	(12)	Resveratrol	HFD-fed mice	Cheng et al. (2019)
	(13)	Celastrol	Free fatty acid (FFA)-treated HepG2 cells	(!!! INVALID CITATION !!!)
	(14)	Isoorientin	high fructose-fed mice	Yuan et al. (2016)
	(15)	Geraniol	high fructose-fed rats; MCD-fed rats	(Ibrahim et al., 2015; Chen et al., 2016)
	(16)	Astaxanthin	high fructose-fed mice	Bhuvaneswari et al. (2014)
	(17)	Schisandrin B	LPS-activated RAW264.7 macrophages	Leong et al. (2016)
	(18)	Kukoamine B	High-fat diet/high-fructose (HFDFr)-fed rats	Zhao et al. (2020b)
	(19)	Genistein	MCD-fed mice	Yoo et al. (2015)
	(20)	Naringenin	MCD-fed mice	Wang et al. (2020)
	(21)	Scopolamine	MCD-fed mice	Liu et al. (2020)
Regulating fibrosis	(22)	Isorhamnetin	Combination of diet and chemical inducers (HFD + CCl4)	Ganbold et al. (2019)
	(23)	Astragaloside	CCl4 induced mouse model	Yuan et al. (2018)
	(24)	Salvianolic acid B	CDAA diet + CCl4	Hong et al. (2017)
	(25)	Pycnogenol	MCD + HFD-fed mice	Mei et al. (2012)
	(26)	Calycosin	MCD-fed mice	Duan et al. (2017)
	(27)	Glycyrrhetinic acid	MCD-fed mice	Wang et al. (2016)
	(28)	Xanthohumol	Paigen diet-fed mice and rats	Dorn et al. (2010)
	(29)	Thymoquinone	HFHC diet-fed	Awad et al. (2016)
	(30)	Isochlorogenic acid B	MCD-fed mice	Liu et al. (2019a)
Regulating Gut Microbiota	(31)	Salidroside	HFD-fed mice	Li et al. (2020b)
	(32)	Quercetin	HFD-fed mice	Porras et al. (2019)
